# Transcriptomic data analysis and differential gene expression of antioxidant pathways in king penguin juveniles (*Aptenodytes patagonicus*) before and after acclimatization to marine life

**DOI:** 10.1016/j.dib.2016.09.021

**Published:** 2016-09-22

**Authors:** Benjamin Rey, Cyril Dégletagne, Claude Duchamp

**Affiliations:** a Université de Lyon, F-69000, Lyon, Université Lyon 1, CNRS - Laboratoire de Biométrie et Biologie Evolutive, F-69622 Villeurbanne Cedex, France; b Université de Lyon, F-69000, Lyon, Université Lyon 1, CNRS - Laboratoire d׳Ecologie des Hydrosystèmes Naturels et Anthropisés, F-69622 Villeurbanne Cedex, France

**Keywords:** Microarray, Penguin, Muscle, Antioxidant pathways

## Abstract

In this article, we present differentially expressed gene profiles in the pectoralis muscle of wild juvenile king penguins that were either naturally acclimated to cold marine environment or experimentally immersed in cold water as compared with penguin juveniles that never experienced cold water immersion. Transcriptomic data were obtained by hybridizing penguins total cDNA on Affymetrix GeneChip Chicken Genome arrays and analyzed using maxRS algorithm*, “*Transcriptome analysis in non-model species: a new method for the analysis of heterologous hybridization on microarrays*”* (Dégletagne et al., 2010) [1]*.* We focused on genes involved in multiple antioxidant pathways. For better clarity, these differentially expressed genes were clustered into six functional groups according to their role in controlling redox homeostasis. The data are related to a comprehensive research study on the ontogeny of antioxidant functions in king penguins, “Hormetic response triggers multifaceted anti-oxidant strategies in immature king penguins (Aptenodytes patagonicus)” (Rey et al., 2016) [2]*.* The raw microarray dataset supporting the present analyses has been deposited at the Gene Expression Omnibus (GEO) repository under accessions GEO: GSE17725 and GEO: GSE82344.

**Specifications Table**

**Table:** 

Subject area	*Biology*
More specific subject area	*Oxidative stress physiology*
Type of data	*Table*
How data was acquired	*Microarray data were obtained by DNA microarray hybridization (Affymetrix GeneChip® Chicken Genome Array). Tissue: pectoralis muscle biopsy of juvenile king penguins excised under general anesthesia.*
Data format	*Analyzed, raw data*
Experimental factors	*Total RNA was extracted from pectoralis muscle; biotin labeling and hybridization were performed following standard Affymetrix protocol.*
Experimental features	*Never-immersed juvenile penguins serve as control and were compared i) to naturally acclimated penguins returning from a foraging trip at sea and ii) to naïve penguins artificially acclimated to cold water by repeated immersions.*
Data source location	*Port Alfred, Possession Island (Crozet Archipelago, 46°25’ S, 51°45’ E) and Lyon University (France).*
Data accessibility	*Data is within this article and raw data is available in Gene Expression Omnibus repositories (GEO:*GSE17725; http://www.ncbi.nlm.nih.gov/geo/query/acc.cgi?acc=GSE17725*and GEO:*GSE82344; http://www.ncbi.nlm.nih.gov/geo/query/acc.cgi?token=yxibwieatzavpqv&acc=GSE82344).

**Value of the data**

• Our transcriptomic analysis of gene expression in the pectoralis muscle of wild juveniles king penguin allows the detection of multiple antioxidant pathways.
• These data provided evidences that an activation of powerful and coordinated antioxidant strategies occurs in the pectoralis muscle of king penguin juveniles during the transition from a terrestrial to a marine life style.
• These original results can serve as a reference point for various studies related to the mechanisms controlling redox homeostasis in natural populations for which data availability remains scarce and usually restricted to the detection of few antioxidant molecules.

## 1 Data

Here, we provide the expression profile of gene involved in the control of redox homeostasis in the pectoralis muscle of three groups of king penguin juveniles (*Aptenodytes patagonicus*) differing in their degree of acclimation to marine environment. Targeted genes are clustered into six groups as follow: the genes encoding proteins involved in non-mitochondrial ROS generation (Cluster 1), antioxydant enzymes (cluster 2), heat choc and chaperone proteins (Cluster 3), DNA repairs processes (Cluster 4), repair or degradation of damaged proteins (Cluster 5) and lipid membrane composition remodeling (Cluster 6). For each gene we provide its symbol, its name, the corresponding Affymetrix ProbeSet identification number and the percentage change of expression as compared to never-immersed control penguins.

## 2 Experimental design, materials and methods

### 2.1 Animals and sample collection

We captured king penguin juveniles of 10–11 month at the breeding colony of la Baie du Marin (Crozet Archipelago; French Southern Territories). A first group of penguins was hold in an outdoor enclosure until they achieved their molt constituting the ‘never-immersed control’ group (NI, *n*=4). A second group of penguins received the same treatment as the NI penguins but were subjected to repeated immersions in cold water (8 °C) over 3 weeks to simulate the acclimatization to marine life; this group is refereed as artificially-acclimated penguins (AA, *n*=3). NI penguins were also compared to juveniles of 12–14 month that returned from a foraging trip at sea and had fully accomplished their acclimatization to marine life (sea-acclimatized, SA, *n*=3). We controlled for potential effect of nutritional status by feeding penguins with mackerel (*Scomber vernalis*) on a daily basis. At the end of the procedure, pectoralis muscle of each penguin was surgically biopsied under general anesthesia and the muscle biopsy was frozen at −80 °C. More details of the experimental procedure are given in Rey et al. [2].

### 2.2 RNA extraction

Total RNA was extracted following the single-step TriReagent protocol (Invitrogen, Cergy Pontoise, France). Briefly, 50 mg of pectoralis muscle was homogenized in 1 mL reagent with a Polytron homogenizer. The aqueous phase was transferred to a 2 mL Eppendorf tube containing 0.5 ml 2-propanol. Samples were incubated at room temperature for 5 min and subjected to a centrifugation at 12,000*g* for 10 min at 4 °C. The pellet was washed twice with ethanol 75% and was re-suspended in ultra-pure water. The quality of extracted RNA (RNA integrity>8) was assessed using a Bioanalyzer 2100 (Agilent technologies, Inc, Palto Alto, CA, USA).

### 2.3 Labeling and hybridization

Labeling and hybridization were performed on Affymetrix GeneChip® Chicken Genome Arrays by the ProfileXpert platform (Lyon, France) following the standard Affymetrix protocol (http://www.affymetrix.com), as described in Dégletagne et al. [1]. All arrays were scanned with a confocal laser (Genechip scanner 3000, Affymetrix).

### 2.4 Microarray analysis

We used the MaxRS method developed for the analysis of heterologous hybridization profiles [1], a method that has been previously applied in king penguins [3]. All results were normalized using the quantile method after log2 transformation to make them comparable across microarrays [4]. Gene expression of NI penguins, considered as control in the study, were compared to those of SA or AA groups. Differentially expressed genes between NI vs. SA or NI vs. AA were determined using the empirical Bayes moderated *t-statistics* implemented in the Bioconductor package limma [5]. We focused on the genes involved in the redox homeostasis and gathered them into six functional clusters according to GenOntology annotation and literature search [2], [6], [7]. All analyses were performed using the *R* statistical software Table 1.

## Conflict of interest

None.

## Figures and Tables

**Table 1:** Microarray data analysis centered on the genes encoding proteins involved in the regulation of the redox homeostasis.

**Symbol**	**Name**	**PPSets**	**log2**	**SA/NI**	***P*- value**	**log2**	**AA/NI**	***P*- value**
			**(SA/NI)**	**%**		**(AA/NI)**	**%**	
**Cluster 1: Genes encoding non mitochondrial proteins involved in Reactive Oxygen Species (ROS) generation**								
ANGPTL4	angiopoietin-like 4	GgaAffx.395.1.S1_at	−0.31	−19%	0.033			ns
AOX1	aldehyde oxidase 1	GgaAffx.5165.3.S1_at	−0.49	−29%	0.031	−0.49	−29%	0.031
AOX2	aldehyde oxidase 2	GgaAffx.5165.4.S1_s_at	0.68	61%	0.003	0.42	34%	0.037
DUOX2	dual oxidase 2	GgaAffx.1631.1.S1_s_at	−0.35	−22%	0.029			ns
DUOXA1	dual oxidase maturation factor 1	GgaAffx.1645.3.S1_s_at	0.28	22%	0.017			ns
NOX1	NADPH oxidase 1	GgaAffx.22036.3.S1_s_at	−0.3	−19%	0.042			ns
PXDN	peroxidasin homolog	Gga.14999.1.S1_at	−1.2	−56%	0.004	−0.94	−48%	0.016
SCARF1	scavenger receptor class F. member 1	Gga.7260.2.S1_at	−0.73	−40%	0.000	−0.42	−25%	0.014
SIRT1	sirtuin 1	GgaAffx.1802.1.S1_at	0.48	39%	0.050			ns
SIRT5	sirtuin 5	Gga.12456.1.S1_at	0.82	77%	0.002			ns
SIRT6	sirtuin 6	GgaAffx.23594.1.S1_at	0.36	28%	0.042			ns
TNFRSF11A	tumor necrosis factor receptor superfamily. member 11a NFKB activator	GgaAffx.8155.1.S1_at	−0.42	−26%	0.024			ns
TNFRSF18	tumor necrosis factor receptor superfamily. member 18	GgaAffx.11426.1.S1_at	−0.51	−30%	0.007			ns
TNFRSF21	tumor necrosis factor receptor superfamily. member 21	Gga.4943.1.S1_at	−1.16	−55%	0.012	−0.79	−43%	0.045
							
**Cluster 2: Genes encoding antioxydant enzymes**								
BLVRA	biliverdin reductase A	GgaAffx.23872.1.S1_at	0.51	42%	0.039			ns
GPX4	glutathione peroxidases	Gga.107.1.S1_at	0.94	92%	0.000			ns
HMOX1	heme oxygenase 1	Gga.2039.1.S1_at	1.39	162%	0.050	2.17	350%	0.006
HMOX2	heme oxygenase (decycling) 2	Gga.9310.1.S1_s_at	−0.57	−33%	0.003			ns
MGST3	microsomal glutathione S-transferase 3	Gga.7258.1.S1_at	0.6	52%	0.010			ns
MT2A	metallothionein 2A	Gga.4210.1.S1_at	2.06	316%	0.001	1.66	217%	0.004
MT3	metallothionein 3	GgaAffx.9262.1.S1_at	1.48	180%	0.005	1.44	171%	0.006
PRDX3	peroxiredoxin 3	Gga.4515.3.S1_a_at	0.42	34%	0.015			ns
SOD1	superoxide dismutase 1	Gga.3346.1.S1_a_at	0.42	34%	0.025			ns
TXNDC10	thioredoxin domain containing 10	Gga.17473.1.S1_s_at	−0.96	−49%	0.000	−0.71	−39%	0.001

**Cluster 3: Genes encoding heat shock or chaperone proteins**								
HSF3	heat shock factor 3	Gga.5116.3.S1_a_at	0.33	26%	0.023			ns
HSF4	heat shock transcription factor 4	GgaAffx.2032.2.S1_s_at	0.45	36%	0.022			ns
CRYAA	crystallin. alpha A	GgaAffx.10353.1.S1_at	0.39	31%	0.027			ns
CRYAB	crystallin. alpha B	Gga.1999.1.S1_a_at	0.96	95%	0.021			ns
HSPE1	heat shock 10 kDa protein 1	Gga.4873.1.S1_a_at	−−0.55	−32%	0.002	−0.33	−20%	0.039
HSPB1	heat shock 27 kDa protein 1	Gga.1809.1.S1_at	−0.45	−27%	0.008			ns
HSPB7	heat shock 27 kDa protein family. member 7	Gga.11398.1.S1_at	0.95	93%	0.000			ns
HSPD1	heat shock 60 kDa protein 1	Gga.9897.1.S1_at	−0.75	−41%	0.000	−0.86	−45%	0.000
DNAJA4	DnaJ (Hsp40) homolog. subfamily A. member 4	Gga.5900.3.S1_a_at	−0.51	−30%	0.010	−0.44	−26%	0.021
DNAJB9	DnaJ (Hsp40) homolog. subfamily B. member 9	GgaAffx.12760.1.S1_s_at	−0.87	−45%	0.019	−1.10	−53%	0.005
DNAJC6	DnaJ (Hsp40) homolog. subfamily C. member 6	GgaAffx.23432.1.S1_s_at	−0.48	−28%	0.004			ns
HSP67B2	similar to heat shock protein 67B2	Gga.16163.1.S1_s_at	1.38	160%	0.000			ns
HSP70	heat shock protein 70	Gga.4942.1.S1_at	−0.88	−46%	0.000	−0.51	−30%	0.016
HSPA14	heat shock 70 kDa protein 14	Gga.19503.1.S1_at	−0.61	−34%	0.001	−0.44	−27%	0.011
HSPA8	heat shock 70 kDa protein 8	Gga.4555.1.S1_a_at	−0.71	−39%	0.003			ns
							
**Cluster 4: Genes encoding proteins involved in DNA repair processes**								
PARP6	poly (ADP-ribose) polymerase family. member 6	Gga.1599.1.S1_s_at	0.29	22%	0.045			ns
PARP8	poly (ADP-ribose) polymerase family. member 8	GgaAffx.24537.1.S1_s_at	0.31	24%	0.040	0.34	27%	0.024
PARP16	poly (ADP-ribose) polymerase family. member 16	Gga.8044.1.S1_at	0.43	35%	0.037			ns
XRCC2	X-ray repair complementing defective repair cells 2	Gga.12290.1.S1_at	−0.55	−32%	0.003			ns
XRCC4	X-ray repair complementing defective repair cells 4	GgaAffx.24733.1.S1_s_at	0.29	22%	0.025			ns
ERCC4	excision repair cross-complementing group 4	GgaAffx.12489.1.A1_at	0.54	45%	0.032			ns
RAD21L1	RAD21-like 1	GgaAffx.3857.1.S1_at	0.45	37%	0.022			ns
RAD51L3	RAD51-like 3	Gga.9680.1.S1_x_at	0.29	22%	0.035			ns
RAD23B	RAD23 homolog B	Gga.1359.1.S1_at	0.31	24%	0.037	0.43	34%	0.008
DDB1	damage-specific DNA binding protein 1. 127 kDa	Gga.5146.1.S1_at	0.49	40%	0.007			ns
DDB2	damage-specific DNA binding protein 2. 48 kDa	GgaAffx.12520.1.S1_s_at	0.27	21%	0.048	0.31	24%	0.031
POLE	polymerase (DNA directed). epsilon	GgaAffx.4785.1.S1_at	−0.44	−26%	0.003	−0.58	−33%	0.000
POLE3	polymerase (DNA directed). epsilon 3	Gga.5487.1.S1_at	−0.62	−35%	0.006			ns
RFC1	replication factor C (activator 1) 1. 145 kDa	GgaAffx.20533.1.S1_s_at	0.91	89%	0.000			ns
UNG	uracil-DNA glycosylase	Gga.4682.1.S1_at	−0.42	−25%	0.036	−0.43	−26%	0.033
MBD4	methyl-CpG binding domain protein 4	Gga.3616.1.S1_at	−0.3	−19%	0.034			ns
							
**Cluster 5: Genes encoding proteins involved repair or degradation of damaged proteins**								
MSRA	methionine sulfoxide reductase A	GgaAffx.25021.1.S1_s_at	0.65	57%	0.001			ns
PSMA7	proteasome subunit. alpha type. 7	Gga.2045.2.S1_a_at	0.58	49%	0.006	0.58	49%	0.006
PSMB1	proteasome subunit. beta type. 1	Gga.4653.2.S1_a_at	0.38	31%	0.043			ns
PSMB3	proteasome subunit. beta type. 3	Gga.1459.1.S1_at	0.52	43%	0.000	0.71	63%	0.000
PSMC3	proteasome 26S subunit. ATPase. 3	Gga.4649.1.S1_s_at	0.36	28%	0.008			ns
PSMC6	proteasome 26S subunit. ATPase. 6	Gga.16005.1.S1_s_at	0.6	52%	0.032			ns
PSMD4	proteasome 26S subunit. non-ATPase. 4	Gga.6030.1.S1_s_at	0.33	26%	0.010			ns
PSME3	proteasome activator subunit 3	Gga.5999.2.S1_at	−0.42	−25%	0.021			ns
–	proteasome C1 subunit	GgaAffx.8554.1.S1_x_at	0.33	26%	0.025	0.45	37%	0.005
POMP	proteasome maturation protein	Gga.5765.1.S1_at	0.32	25%	0.020	0.44	36%	0.003
SMURF1	SMAD specific E3 ubiquitin protein ligase 1	GgaAffx.2883.1.S1_s_at	0.81	75%	0.011			ns
UBB	ubiquitin B	Gga.2501.2.S1_at	0.41	33%	0.023			ns
UBE2G2	ubiquitin-conjugating enzyme E2G 2	Gga.19739.1.S1_at	0.38	30%	0.003			ns
UBE4B	ubiquitination factor E4B	GgaAffx.25563.1.S1_s_at	0.39	31%	0.014			ns
UCHL1	ubiquitin carboxyl-terminal esterase L1	Gga.9618.1.S1_at	2.22	366%	0.000	1.84	258%	0.000
UCHL5	ubiquitin carboxyl-terminal hydrolase L5	GgaAffx.12236.1.S1_s_at	0.61	52%	0.029			ns
UFD1L	ubiquitin fusion degradation 1 like	Gga.3094.1.S1_at	0.44	36%	0.010			ns
UIMC1	ubiquitin interaction motif containing 1	GgaAffx.768.2.S1_at	0.67	59%	0.001			ns
WWP1	WW domain containing E3 ubiquitin protein ligase 1	GgaAffx.24796.1.S1_at	0.45	37%	0.022			ns
LONP2	lon peptidase 2. peroxisomal	Gga.12947.1.S1_s_at	0.68	60%	0.000			ns
LONRF1	LON peptidase N-terminal domain and ring finger 1	GgaAffx.8741.1.S1_at	0.31	24%	0.041			ns
ATXN3	ataxin 3	Gga.12408.1.S2_at	−0.85	−44%	0.000	−0.48	−29%	0.016
NBR1	neighbor of BRCA1 gene 1	Gga.9984.1.S1_s_at	0.43	34%	0.013			ns
							
**Cluster 6: Genes encoding proteins involved in lipid membrane composition**								
MBOAT2	membrane bound O-acyltransferase 2	GgaAffx.10502.2.S1_s_at	0.92	89%	0.027			ns
SCD5	stearoyl-CoA desaturase 5	Gga.6052.3.S1_a_at	0.36	28%	0.048	0.41	33%	0.028

Differentially expressed genes are presented as percentage change of never-immersed (NI) controls versus naturally acclimated to cold marine environment (sea acclimated: SA) or experimentally immersed in cold water (artificially acclimated: AA). For each gene, we provided its symbol followed by its common name and the Affymetrix ProbeSet identification number used to measure its expression. Genes were considered significantly differentially expressed when *p*-value <0.05.
